# Modeling myeloid cell development in health and disease using induced pluripotent stem cells

**DOI:** 10.3389/fimmu.2026.1815809

**Published:** 2026-07-15

**Authors:** Ivan Tesakov, Masoud Nasri, Maksim Klimiankou, Betül Findik, Peter Loskill, Julia Skokowa

**Affiliations:** 1 Department of Oncology, Hematology, Clinical Immunology, and Rheumatology, University Hospital Tuebingen, Tuebingen, Germany; 2 Institute of Biomedical Engineering, Eberhard Karls University Tuebingen, Tuebingen, Germany; 3 NMI Natural and Medical Sciences Institute at the University of Tuebingen, Reutlingen, Germany

**Keywords:** bone marrow failure, induced pluripotent stem cells, leukemia, model systems, myeloid malignancies, myelopoiesis

## Abstract

The pioneering discovery by Yamanaka and colleagues enabling the reprogramming of terminally differentiated somatic cells into induced pluripotent stem cells (iPSCs) has opened transformative opportunities for disease modeling and regenerative medicine, particularly in the context of inherited monogenic disorders. Patient-specific iPSCs can be generated, expanded almost indefinitely, and differentiated into a broad spectrum of cell types, including hematopoietic stem and progenitor cells, mature myeloid cells, and leukemic cells. Despite important limitations – such as epigenetic memory, variable differentiation efficiency, and concerns regarding tumorigenicity – iPSCs have become an indispensable experimental platform for studying inherited hematological disorders and malignancies, providing a renewable and physiologically relevant source of cells for downstream analyses. Beyond their research applications, iPSC-derived blood cells are increasingly being explored in preclinical studies and early-phase clinical trials as potential therapeutic products. The advent of CRISPR/Cas9 genome editing, pioneered by Charpentier and Doudna, has further advanced iPSC-based models by enabling precise correction or introduction of disease-causing mutations and the generation of isogenic control lines. This approach facilitates detailed mechanistic studies of defective hematopoiesis, enables drug discovery and repurposing through *in silico* screening platforms – such as L1000CDS^2^ and the Connectivity Map – and supports preclinical therapeutic validation. In this review, we summarize key applications of iPSC technology in hemato-oncology, discuss its major advantages and current limitations, and highlight emerging directions, including scalable iPSC-derived blood cell therapies for inherited and acquired bone marrow failure syndromes and leukemia.

## Introduction

Historically, studies of the pathogenesis of hematological disorders have been limited by the availability of patient-derived tissues. Relying on primary human cells poses significant challenges, including limited sample availability, ethical concerns regarding repeated bone marrow biopsies to obtain hematopoietic stem cells, and the rapid exhaustion or phenotypic drift of primary hematopoietic stem and progenitor cells (HSPCs) in culture (1). Moreover, although murine models have been invaluable, the significant differences in the ontogeny of human versus murine tissues can result in imperfect recapitulation of human disease phenotypes, particularly in complex myeloid malignancies or bone marrow failure syndromes (BMFSs) (2). In addition to mice, zebrafish have emerged as a complementary *in vivo* model for BMFSs, offering advantages such as rapid development, transparent embryos for real-time observation of hematopoiesis, and faithful phenocopying of key inherited syndromes like Fanconi anemia, Diamond-Blackfan anemia, severe congenital neutropenia (CN), dyskeratosis congenita, and Shwachman-Diamond syndrome (3–5). While zebrafish offer these advantages, iPSC technology is often superior due to its human-specific genetic fidelity, indefinite expandability, and ability to faithfully capture patient-specific clonal architecture (6). These limitations have necessitated the adoption of induced pluripotent stem cell (iPSC) technology. iPSCs bridge the gap between primary samples and various model systems by providing an indefinitely scalable and genetically faithful platform. A critical advantage of this technology is its ability to capture and preserve the clonal architecture of heterogeneous blood cancers. For instance, reprogramming patient samples allows for the isolation of subclones representing distinct stages of leukemic evolution – from pre-leukemic clonal hematopoiesis to frank leukemia – within a single patient background (7).

## Generation and maintenance of iPSCs for hematological research

The seminal work by Takahashi and Yamanaka demonstrated that iPSCs can be generated by reprogramming somatic cells into a pluripotent state that closely resembles embryonic stem cells (ESCs), most commonly through ectopic expression of defined transcription factors. For the first time, they found that retroviral delivery of four transcription factors – Oct3/4, Sox2, Klf4, and c-Myc (OSKM) – into mouse (8) and human (9) fibroblasts is sufficient to generate pluripotent cell colonies with ESC-like morphology, transcriptomic profiles, and differentiation potential (8, 9). These studies fundamentally changed the view of somatic cell identity by showing that it is reversible rather than fixed. They established iPSCs as a renewable human cell platform that enables derivation of hematopoietic and myeloid lineages (among others) for both mechanistic studies and disease modeling.

### Reprogramming factors and mechanisms

Oct4 and Sox2 form the core of the pluripotency transcriptional network by co-occupying enhancers and promoters of pluripotency genes while actively repressing somatic transcriptional programs. In contrast, Klf4 and c-Myc primarily promote proliferation, chromatin accessibility, and metabolic remodeling of cells (8–10). Early during reprogramming, OSKM binding triggers downregulation of mesenchymal gene expression and induction of epithelial features, followed by widespread chromatin remodeling with loss of lineage-specific DNA methylation and gradual activation of endogenous pluripotency gene networks (10). Subsequent work showed that c-Myc is dispensable for iPSC generation, although its omission results in reduced efficiency and slower reprogramming kinetics (11). In mouse fibroblasts, exclusion of Oct4 and reprogramming with Sox2, Klf4 and c-Myc (SKM) is also sufficient to generate iPSCs that display ESC-like morphology, reactivate endogenous pluripotency genes, form teratomas, and support full-term development in tetraploid complementation assays (12). However, most currently used protocols for reprogramming of human cells rely on the full OSKM set, and studies manipulating relative factor levels indicate that their stoichiometry and temporal dynamics are critical determinants of both reprogramming efficiency and the molecular properties of the resulting iPSC lines (13).

### Somatic cell sources and delivery systems

A wide range of somatic cell types can be used as starting material for iPSC generation. This includes dermal fibroblasts, keratinocytes, peripheral blood mononuclear cells (PBMCs), CD34^+^ HSPCs, and urine-derived epithelial cells (9, 11, 14–16). CD34^+^ cells from peripheral blood are particularly attractive for hematology and myeloid disease modeling because they are easily accessible, already committed to the hematopoietic lineage, and often harbor disease-defining somatic mutations in patients with myeloid malignancies (17–19). Erythroblasts expanded *ex vivo* from small volumes of peripheral blood can also serve as highly reprogrammable substrates, particularly when sample material is limited (20, 21).

The method used to deliver reprogramming factors has evolved substantially since the first reports. Integrating viral vectors, such as retro- and lentiviruses, were initially favored because of their high efficiency in transducing somatic cells and robust induction of pluripotency. They are still broadly used for basic research applications (8, 9, 22). However, integration of reprogramming cassettes into the host genome raises concerns about insertional mutagenesis, transgene reactivation, and interference with downstream lineage commitment (23, 24). To overcome these limitations, multiple non-integrating approaches have been developed, including Sendai virus vectors (25, 26), episomal plasmids (17, 27), synthetic mRNAs (28), and recombinant proteins (29). Among these, Sendai virus-based systems have become widely adopted for generating disease-specific and clinical-grade iPSC lines from blood-derived cells (25, 30). Because Sendai virus replicates only in the cytoplasm and is lost during cell passaging, it combines high reprogramming efficiency with the production of transgene-free iPSCs (25, 26, 30). Episomal plasmid approaches offer an alternative integration-free strategy and have been successfully applied to blood- and cord blood-derived cells, although reprogramming efficiencies are generally lower than with viral systems (17, 28).

### Pluripotency networks and epigenetic resetting

Successful reprogramming requires adequate re-establishment of the pluripotency gene regulatory network as well as the epigenetic landscape, triggered by ectopically expressed OSKM transcription factors. OCT4, SOX2, and NANOG form an interconnected autoregulatory circuit that maintains pluripotency by activating downstream targets such as LIN28, DPPA4, and UTF1 while repressing differentiation-associated genes (31, 32). Chromatin remodelers and epigenetic modifiers, including TET enzymes, Polycomb complexes, and histone acetyltransferases, cooperate with OSKM to erase somatic DNA methylation patterns and to re-establish bivalent chromatin domains at key developmental loci (33–35).

However, reprogramming might be incomplete and can leave residual epigenetic memory that biases the differentiation potential of iPSCs in a tissue-of-origin-dependent manner (36, 37). In the context of myeloid disease modeling using patient-derived iPSCs from malignant or pre-malignant hematopoietic cells, extensive chromatin remodeling during reprogramming can erase disease-associated epigenetic and transcriptional signatures, which can be re-established upon differentiation into hematopoietic (and subsequent myeloid) lineages (6, 7, 38). This “epigenetic reset” followed by lineage-specific re-imprinting is a key concept when evaluating disease phenotypes in iPSC-derived myeloid cells (19, 39).

### Culture systems for iPSC maintenance

Maintaining iPSCs in a stable, undifferentiated state is essential for reproducible differentiation into hematopoietic and myeloid lineages. Early protocols used mouse embryonic fibroblast (MEF) feeder layers and serum-containing media supplemented with basic fibroblast growth factor (bFGF) to support iPSC self-renewal and suppress spontaneous differentiation (9, 40, 41). Feeder-based systems remain robust and are still widely used for large-scale differentiation, including protocols for iPSC-derived macrophages and granulocytes in which irradiated MEFs or feeder cells provide trophic support before hematopoietic induction (42, 43). However, feeder-based culture introduces xenogeneic mouse components, which can complicate downstream molecular profiling due to cross-species contamination (44). Moreover, the presence of animal-derived components is a major barrier for translational or GMP-oriented applications (45).

To reduce xenogeneic components and facilitate further clinical translation, feeder-free systems have been developed using defined extracellular matrices (for example vitronectin, laminin-521, Matrigel) and chemically defined media such as mTeSR1, E8 or similar formulations (46–49). These conditions enable expansion of iPSCs and are compatible with monolayer-based hematopoietic differentiation methods, including protocols for generation of neutrophils and macrophages (50–53). Regardless of the culture system, iPSC maintenance requires control of iPSC colony morphology, regular passaging to avoid over-confluence, and regular monitoring of pluripotency marker expression (e.g., OCT4, SOX2, NANOG, TRA-1-60, SSEA-4) as well as genomic integrity by karyotyping or high-resolution sequencing methods (54–58). The International Society for Stem Cell Research (ISSCR) has developed guidelines and standards for human iPSC research, including recommended characterization, quality control, and transparent reporting practices (59).

### Genetic stability and line-to-line variability

Genetic instability is a major concern for long-term iPSC culture and disease modeling, particularly for myeloid malignancies, which are themselves driven by clonal evolution, triggered by chromosomal aberrations or gene mutations. iPSCs can acquire recurrent chromosomal abnormalities (for example, 12p amplifications) and copy-number variants that may confer a proliferative advantage on iPSCs and alter differentiation propensity (60–63). For myeloid disease modeling, comprehensive genomic characterization is critical to ensure that iPSC lines capture, for example, the patient’s genotype (inherited mutations) as well as leukemic or pre-leukemic clones without additional driver mutations introduced during reprogramming or expansion (7, 64, 65).

Substantial line-to-line variability in differentiation potential can arise from genetic background, residual epigenetic memory, and the reprogramming procedure (36, 66–68). Large-scale comparative studies indicate that donor-specific genetic variation is the main source of phenotypic heterogeneity, with epigenetic and technical factors contributing to additional variance (68–71). In the context of myeloid differentiation, this variability manifests as differences in the efficiency of hematopoietic specification, colony-forming output, lineage bias (for example, granulocytic vs. monocytic), and functional responses (cytokine signaling, oxidative burst, etc.) (42, 51, 72, 73).

Taken together, robust iPSC generation and maintenance are essential, but often neglected, prerequisites for interpretable myeloid disease models. Non-integrating reprogramming, defined culture conditions, and systematic phenotypic and genetic quality control of iPSCs can reduce technical variability and help preserve their genetic features (7, 56, 71, 74).

## Hematopoietic and myeloid differentiation of iPSCs

### Hematopoietic differentiation of iPSCs: general principles

Differentiation of human iPSCs into hematopoietic and myeloid cells follows developmental logic, recapitulating mesoderm induction, hemato-endothelial specification, and emergence of CD34^+^CD43^+^ or CD34^+^CD45^+^ hematopoietic progenitors, which can further be directed into myeloid lineages. The methods of hematopoietic differentiation of iPSCs were initially established using human embryonic stem cells (hESCs). Kennedy et al. demonstrated that hESC differentiation cultures mirror the early stages of embryonic hematopoietic development, with a “hemangioblast” population emerging first, which subsequently gives rise to both endothelial and hematopoietic lineages (75). Parallel work in mouse ESCs showed that stepwise specification toward hematopoietic fate is driven by sequential exposure to bone morphogenetic protein-4 (BMP4), activin A, bFGF, and vascular endothelial growth factor (VEGF), alone or in combination, with each factor acting at defined developmental windows (76). These principles were subsequently applied to human iPSCs, which displayed comparable hematopoietic and endothelial differentiation potential to hESCs when subjected to similar protocols (77).

Most current protocols for hematopoietic differentiation of iPSCs employ a staged sequence of growth factors and small molecules that recapitulate embryonic hematopoietic ontogeny. These can be grouped into the following functional phases:

#### I. Mesoderm induction (days 0–2)

BMP4 is the principal driver, with short-term high-concentration treatment promoting primitive streak and mesoderm formation. Prolonged or excessive exposure to BMP4 can divert cells toward trophoblast or extra-embryonic endoderm (78). Canonical Wnt/β-catenin signaling, typically activated via CHIR99021, in combination with BMP4, promotes mesoderm commitment (79, 80). Activin A can further support hematopoietic-fated mesoderm formation by inducing *TBXT* expression (81).

#### II. Hemato-endothelial specification (days 2–5)

VEGF is essential for the emergence of KDR^+^ hemogenic endothelium and early hematopoietic progenitors, while bFGF supports proliferation of these populations (76). Subsequent endothelial-to-hematopoietic transition (EHT) critically depends on interleukin-3 (IL-3). Ackermann et al. showed that IL-3 is necessary to drive EHT and generate myeloid progenitors from iPSC-derived hemato-endothelial cells (82).

#### III. Hematopoietic progenitor expansion and myeloid specification (days 5–12+)

Following EHT, the emerging CD34^+^ hematopoietic progenitors require cytokine support for survival, self-renewal, and expansion. Stem cell factor (SCF) and FLT3 ligand (FLT3L) expand CD34^+^ progenitors by inducing quiescent cells to enter the cell cycle (83, 84). Thrombopoietin (TPO) maintains the balance between self-renewal and lineage commitment across erythroid, megakaryocytic, and myeloid compartments (85, 86). Interleukin-6 (IL-6) can act synergistically to further enhance expansion of multipotent progenitors (87).

For protocols aimed at myeloid cell generation, granulocyte colony-stimulating factor (G-CSF) or macrophage colony-stimulating factor (M-CSF) can already be introduced during the expansion phase to bias differentiation toward granulocytic or monocytic lineages, respectively (42). G-CSF can induce proliferation of granulocyte-committed progenitors and promote neutrophil maturation, whereas M-CSF drives monocyte/macrophage commitment (88–90). The choice, dosage and timing of adding lineage-specific cytokines to the iPSC culture determine the final myeloid cell type generated from iPSC-derived hematopoietic progenitors. These general principles are implemented through several major protocol formats, summarized in the following section.

### Existing hematopoietic and myeloid differentiation protocols

A variety of protocols have been developed to differentiate iPSCs into hematopoietic and downstream myeloid lineages, differing in culture format, cytokine regimens, and the use of feeder cells or serum. These can be broadly grouped into four methodological families: stromal co-culture systems, embryoid body (EB)-based spontaneous differentiation, EB-based factor-assisted differentiation, and two-dimensional monolayer systems (88). In addition, transcription-factor-mediated forward programming (e.g., ETV2-based hemato-endothelial programming) has emerged as a complementary route to hematopoietic progenitors (91, 92), but is less well established for myeloid differentiation. Each approach offers distinct trade-offs in efficiency, reproducibility, cost, and suitability for specific downstream applications.

#### I. Stromal (feeder cell) co-culture systems

Stromal co-culture systems rely on direct contact between iPSCs and supportive feeder cells that provide cell-cell interactions, extracellular matrix components, and paracrine signals for hematopoietic specification.

The OP9 mouse bone marrow stromal cell line, derived from osteopetrotic *op/op* mice lacking functional M-CSF production (93, 94), has been extensively used for hematopoietic differentiation of human iPSCs. In the protocol developed by Choi and colleagues, undifferentiated iPSC colonies are placed onto overgrown OP9 monolayers and cultured for 8–9 days, during which mesodermal specification and emergence of CD34^+^CD43^+^ hematopoietic progenitors occur. Lin^-^CD34^+^CD43^+^CD45^+^ cells with robust myeloid colony-forming potential arise by day 6 and peak over the following 2–3 days (95). The resulting progenitors can be further expanded with high-dose GM-CSF. From these expanded progenitors, mature myeloid cells can be generated using lineage-specific cytokine combinations: neutrophils (G-CSF), eosinophils (IL-3 and IL-5), macrophages (M-CSF and IL-1β), dendritic cells (GM-CSF, IL-4, and TNF-α), Langerhans cells (GM-CSF, TGF-β1, and TNF-α), and osteoclasts (GM-CSF, vitamin D_3_, and RANKL) (95). OP9 co-culture has also been shown to efficiently support generation of red blood cells (96) and megakaryocytes/platelets (97) from human iPSCs.

An alternative OP9-based approach specifically optimized for neutrophil generation was developed by Morishima et al., who transferred iPSCs onto OP9 monolayers in the presence of VEGF for 10 days, sorted TRA-1-85^+^CD34^+^VEGFR-2^high^ hemo-angiogenic progenitors, and then co-cultured these with fresh OP9 cells supplemented with IL-3, SCF, and TPO for 20 days followed by a switch to IL-3 and G-CSF for a final 10 days (98). In this system, human iPSC-derived neutrophils acquired key effector functions, including chemotaxis, phagocytosis, and superoxide production, closely resembling peripheral blood neutrophils (98). A similar strategy was independently described by Yokoyama et al. for hESC-derived neutrophils, yielding CD16^+^CD11b^+^ mature neutrophils within 19–31 days (99). These OP9-based systems have proven valuable for modeling congenital neutrophil disorders. For example, Brault et al. applied an optimized OP9-based protocol to iPSCs from patients with *CYBB*-, *CYBA*-, and *NCF1*-related chronic granulomatous disease to generate neutrophils and macrophages that faithfully reproduced the NADPH oxidase deficiency (100).

A derivative line, OP9-DL1, ectopically expresses the Notch ligand Delta-like 1 (DLL1), redirecting differentiation toward T-cell and natural killer (NK)-cell lineages (101). This system has been instrumental for generating iPSC-derived T-cells for immunotherapy applications (102). Notch signaling via OP9-DL1 has also been shown to support generation of dendritic cells from iPSC-derived progenitors (103, 104).

*Advantages and limitations.* OP9-based systems offer several key benefits for myeloid differentiation studies. First, efficient hematopoietic induction is achieved within a short time frame (8–9 days) without the need for exogenous cytokines during the initial co-culture phase, which simplifies the protocol and reduces costs (95). Second, these systems robustly support multilineage output, including not only granulocytes, monocytes, and macrophages but also erythroid, megakaryocytic, and – via OP9-DL1 – lymphoid lineages, enabling comprehensive studies of hematopoietic development from a single platform (94, 96, 102). Third, the differentiation kinetics are well characterized and highly reproducible across multiple iPSC/ESC lines when optimal plating densities and OP9 culture conditions are maintained (77, 95).

Despite these strengths, OP9 co-culture systems have several important drawbacks. First, the serum-containing conditions and undefined feeder-derived factors limit mechanistic dissection of specific signaling pathways controlling hematopoietic specification and myeloid commitment and therefore represent a critical limitation for developmental studies (95). Second, the presence of mouse feeder cells complicates downstream molecular analyses (e.g., multiomics to identify disease-specific deregulated pathways) (44, 105). Third, the use of xenogeneic mouse stromal cells introduces batch-to-batch variability, as the quality and hematopoiesis-inductive capacity of OP9 feeders can decline with passage or inappropriate culture conditions (95). Finally, clinical translation is hindered by the xenogeneic components, which makes current OP9-based protocols unsuitable for manufacturing cell products intended for therapeutic use (51).

#### II. Embryoid body-based spontaneous differentiation

EB-based spontaneous differentiation refers to conditions where iPSCs are aggregated into 3D EBs and allowed to mature without exogenous lineage-directing cytokine cocktails, typically in serum-containing or otherwise permissive media, so that intrinsic patterning cues drive the emergence of derivatives of all three germ layers (106–108).

The approach was first characterized in hESCs: Zambidis et al. demonstrated that hESC-derived EBs cultured in serum-containing medium without recombinant hematopoietic cytokines spontaneously pass EHT and generate sequential waves of primitive and definitive erythro-myeloid progenitors with multilineage colony-forming potential (109). Kyttälä et al. extended this to iPSCs, subjecting genetically matched lines from multiple donors to 21-day spontaneous EB differentiation, showing that all lines produced three-germ-layer derivatives, but donor-specific transcriptional signatures were strongly visible, indicating that the myeloid bias of spontaneous EB hematopoiesis is strongly influenced by genetic background (110).

*Advantages and limitations.* In the iPSC field, truly spontaneous EB-based differentiation has remained a benchmarking tool rather than a scalable production platform. Most detailed mechanistic and functional studies of iPSC-derived myelopoiesis have been performed in guided EB or monolayer systems with defined cytokine cocktails rather than under purely spontaneous conditions (111, 112). Accordingly, the primary utility of spontaneous differentiation lies in providing an unbiased readout of an iPSC line’s intrinsic three-germ-layer competence, which is valuable for line characterization and quality control (110, 113, 114). Its major limitations for hematopoietic applications are very low and variable myeloid yield, poor control over EB internal microenvironment, and pronounced donor-to-donor and clone-to-clone heterogeneity (110). For these reasons, EB-based protocols intended to generate defined hematopoietic or myeloid populations from iPSCs almost invariably rely on guided approaches, which are discussed in the subsequent section.

#### III. Embryoid body-based factor-assisted differentiation

In factor-assisted (also termed guided or directed) EB-based protocols, iPSCs are first aggregated into EBs, but mesoderm induction and hematopoietic specification are actively directed by sequential addition of defined cytokines and growth factors rather than being left to intrinsic patterning. This strategy retains the three-dimensional self-organization of EBs while imposing temporal control over key signaling pathways to enhance the yield and reproducibility of iPSC-derived hematopoietic and myeloid cells (115–117).

The most widely adopted format to generate EBs from iPSCs is the spin EB (forced aggregation) method, in which defined numbers of dissociated iPSCs are centrifuged into V-bottom 96-well plates in a defined serum-free differentiation medium, together with ROCK inhibitor, BMP4, and often VEGF and/or bFGF to drive mesoderm commitment during the first 2–4 days (74, 108, 118, 119). During mesoderm induction, Wnt pathway manipulation has emerged as a key determinant of primitive versus definitive hematopoietic output: GSK3β inhibition can pattern mesoderm toward a definitive hematopoietic program, whereas Wnt inhibition favors primitive hematopoiesis (79).

Following mesoderm induction, EBs are typically plated onto Geltrex- or Matrigel-coated surfaces in a serum-free differentiation medium containing VEGF and SCF, with or without IL-3, to promote EHT and the outgrowth of hemogenic endothelial cells (43, 74, 120). Floating CD34^+^CD45^+^ hematopoietic stem and progenitor cells (HSPCs) typically emerge between days 10 and 14 and can be harvested for further differentiation and analysis (43, 72, 74).

For directed granulopoiesis, EB-derived HPCs can be further cultured with lineage-instructive cytokines. Sweeney et al. described a 32-day protocol combining EB formation, HSPC expansion, and terminal G-CSF-driven maturation (72). Dannenmann et al. applied a similar approach specifically to model CN and demonstrated that iPSC-derived cells from CN patients faithfully recapitulate the maturation arrest of granulopoiesis observed in primary bone marrow and exhibit elevated unfolded protein response and susceptibility to DNA damage (74).

Guided EB-based protocols have also become the most common route to iPSC-derived macrophages (iMacs). In the widely used “monocyte/macrophage factory” approach, pioneered by van Wilgenburg et al., spin EBs are formed in a serum-free differentiation medium supplemented with BMP4, VEGF, and SCF for 4 days to specify hematopoietic mesoderm (121). EBs are then transferred to plates and cultured in medium containing only IL-3 and M-CSF for directed monocytic differentiation. Within approximately two weeks, adherent structures begin to release non-adherent monocyte-like CD14^+^CD16^low^CD163^+^ cells that can be harvested weekly for up to several months and terminally differentiated into mature macrophages by culture with M-CSF alone (121). Scalability of this approach has been further advanced by Ackermann et al., who adapted the EB-based macrophage protocol to stirred-tank bioreactors (122, 123).

*Advantages and limitations*. Guided EB-based protocols combine the physiological benefits of 3D organization – which facilitates niche-like cell-cell interactions, paracrine signaling, and the emergence of hemogenic endothelium – with the reproducibility due to using defined cytokine schedules and standardized EB size (43, 74, 107). Most guided EB-based protocols now employ fully defined, serum-free and xeno-free media, which is advantageous for mechanistic studies, standardization across laboratories, and future clinical translation (43, 124, 125). They allow EB-derived CD34^+^ progenitors to be flexibly directed toward different myeloid lineages by changing cytokine conditions, and the “factory” format can enable scalable production of iPSC-derived macrophages from a single differentiation run (43, 121, 122).

However, guided EB-based methods have notable limitations. They remain labor-intensive due to the requirements for EB formation, plating, and repeated manual harvesting of floating cells. EB-to-EB variability persists even with spin EB standardization, as differences in aggregate compaction and local cytokine gradients within and between EBs introduce heterogeneity in differentiation outcomes (51, 73). Additionally, the myeloid cells generated through EB-based hematopoiesis ontogenetically often resemble yolk-sac-derived tissue-resident macrophages rather than definitive myeloid cells, which may limit their suitability for applications that specifically require adult bone-marrow-derived myeloid identity (112).

#### IV. Two-dimensional monolayer systems

Two-dimensional (2D) monolayer protocols eliminate the need for both feeder cells and three-dimensional EB formation by differentiating iPSCs directly on matrix-coated surfaces through sequential exposure to defined cytokines and small molecules. This approach was pioneered by Niwa et al., who established a serum-free monolayer culture in which human ESCs/iPSCs were guided through three sequential stages on Matrigel-coated dishes: BMP4-driven primitive-streak induction, VEGF/SCF-mediated specification of hemo-angiogenic progenitors, and further lineage-directed differentiation, yielding functional erythrocytes and mature neutrophils, in the absence of feeder cells or serum (126).

In parallel, Salvagiotto et al. developed a feeder-free, serum-free monolayer protocol with BMP4/bFGF/VEGF for mesoderm and hemato-endothelial induction, changed to TPO, SCF, FLT3L, IL-3, and IL-6 to drive hematopoietic differentiation, and followed by G-CSF alone for terminal granulocytic maturation. Under these conditions, they obtained granulocytic cells with CD15 and CD66b expression, demonstrating neutrophil differentiation in a fully 2D, stromal-free system (50).

Yanagimachi et al. later extended the monolayer approach specifically toward monocytic lineage output (127). In their protocol, BMP4-induced primitive-streak cells were sequentially directed through hemo-angioblast specification, hematopoietic expansion, and monocyte-directed differentiation. iPSC-derived monocytes could further be terminally differentiated into functional M1/M2-polarizable macrophages or mature dendritic cells (127). Monkley et al. refined this protocol to achieve higher monocyte yields in a smaller culture format, and demonstrated that iPSC-derived monocytes, macrophages, and dendritic cells undergo a complete transcriptomic switch from pluripotency to lineage-appropriate gene expression profiles comparable to their primary counterparts (128).

More recent 2D protocols have introduced the GSK3β inhibitor CHIR99021 during the initial mesoderm induction. Takata et al. employed a CHIR99021/BMP4/VEGF-based monolayer protocol to generate iPSC-derived iMacs that ontogenetically resemble yolk-sac-derived tissue-resident macrophages; upon exposure to organ-specific cues, these iMacs could be further specified into microglia-like, alveolar macrophage-like, or Kupffer cell-like populations (129).

Tursky et al. performed a direct comparison of four serum-free, feeder-free hematopoietic differentiation methods (two monolayer-based and two EB-based) and demonstrated that an optimized 2D multi-step protocol incorporating extended Wnt activation and aryl hydrocarbon receptor (AhR) hyper-stimulation was the most efficient strategy (51).

Klepikova et al. compared macrophages generated by a 2D monolayer versus an EB-based protocol and found the 2D approach to be more reliable, with no failed differentiations compared with ~27% failures in the EB-based format (73). Although iMacs from both systems showed similar surface phenotype and phagocytic activity, EB-derived cells over-expressed inflammatory response genes, whereas monolayer-derived iMacs were enriched for anti-inflammatory/M2 polarization, antigen presentation, and lipid homeostasis pathways (73).

*Advantages and limitations*. Monolayer protocols provide defined, serum-free, feeder-free conditions that reduce xenogeneic contaminants and improve standardization, which makes them attractive for mechanistic studies, disease modeling, and future GMP translation (126, 127).

However, these protocols usually necessitate more frequent medium and cytokine exchanges (typically every 1–2 days), which can increase hands-on time and reagent consumption compared with simpler EB-based or stromal co-culture approaches in many laboratory implementations (50, 126, 127). In direct side-by-side comparisons of representative serum- and feeder-free methods, an optimized multistep 2D protocol could match or outperform selected EB-based approaches in terms of CD34^+^ output and cost (51), but other monolayer protocols have been reported to generate lower numbers of hematopoietic cells and progenitors than EB-based differentiation systems (130). At the same time, 2D cultures typically exhibit weaker endogenous Wnt/β-catenin activation and reduced responsiveness to BMP4, which can limit mesoderm formation and clonogenic CD34^+^ progenitor output unless compensated by exogenous Wnt agonists (131). Like EB-based protocols, most monolayer systems remain sensitive to iPSC line-dependent variability in terms of cytokine responsiveness and differentiation efficiency (51, 73, 127).

#### V. Transcription factor-mediated forward programming

An alternative to cytokine-driven differentiation is transcription factor (TF)-mediated forward programming, in which forced expression of hematopoietic master regulators directly converts iPSCs into hemato-endothelial or myeloid progenitors.

Elcheva et al. first demonstrated that overexpression of two TFs – *ETV2* and *GATA2* – is sufficient to directly induce a pan-myeloid hemato-endothelial program from iPSCs, whereas GATA2 and TAL1 specify an erythro-megakaryocytic program (132). Lange et al. subsequently established a doxycycline-inducible lentiviral vector-based system in which regulated co-expression of *SCL, LMO2, GATA2,* and *ETV2* (SLGE) drives iPSCs through mesodermal priming, hemato-endothelial specification, and EHT to yield CD34^+^CD45^+^ hematopoietic progenitors with multilineage colony-forming potential (92).

Applying this concept specifically to neutrophil generation, Majumder et al. used transient delivery of ETV2 modified mRNA to induce hemato-endothelial progenitors from iPSCs, expanded them with GM-CSF, bFGF, and UM171, and then terminally matured them with G-CSF and the retinoic acid agonist Am580 into functional neutrophils (91).

*Advantages and limitations.* Forward programming bypasses the slow, multistep cytokine-driven differentiation process and enables rapid generation of relatively homogeneous hemato-endothelial and myeloid progenitor populations with good reproducibility across multiple iPSC lines (92). However, most systems rely on integrating lentiviral vectors that introduce permanent genomic insertions with attendant risks of insertional mutagenesis, transcriptional interference, and transgene silencing, complicating direct clinical translation (92, 133). In practice, forward-programmed cultures often generate relatively low numbers of hematopoietic progenitors (92, 133). Forward-programmed hematopoietic cells so far show minimal durable *in vivo* engraftment and a bias toward primitive rather than definitive hematopoiesis, and these strategies are therefore less widely adopted and less extensively validated for myeloid disease modeling than conventional cytokine-directed methods (92, 134, 135).

### Protocol choice, existing limitations, and future directions

The choice of an appropriate differentiation strategy is a critical determinant of how accurately iPSC-derived hematopoietic cells recapitulate developmental stage, lineage specification, and disease-relevant phenotypes. From a practical perspective, these protocols differ in their suitability for disease modeling and translational applications. Stromal co-culture systems remain efficient for robust multilineage hematopoietic induction and generation of mature myeloid cells, but depend on xenogeneic feeders and serum, which complicates molecular analyses and limits direct translational use (44, 51, 95). Guided EB-based protocols can provide higher yields and support long-term myeloid cell production, including bioreactor-based scaling, but remain labor-intensive and often retain a yolk-sac-like or primitive myeloid bias, requiring careful validation when modeling conditions directly linked to the adult bone marrow-derived myeloid cells or myeloid malignancies (72, 112, 121–123, 129). In contrast, 2D monolayer systems offer defined, feeder-free conditions that are better suited to standardization and GMP-oriented optimization, although efficiency, maturation, and functional output remain highly protocol- and iPSC line-dependent (50, 51, 73, 126–129, 136). Transcription factor-based forward programming offers rapid and relatively homogeneous production of specific progenitor or myeloid-like populations, but bypasses early developmental checkpoints and is less well established for applications that require fully physiological hematopoietic cell maturation or clinical translation (91, 92). Overall, feeder- and serum-free guided EB-based or monolayer systems appear better suited to standardization and large-scale manufacture than feeder-based approaches, although modeling adult myeloid malignancies and clonal hematopoiesis may still require protocols that more efficiently generate definitive hemogenic endothelium and hematopoietic stem cell (HSC)-like progenitors (105, 107, 137).

Across all protocol families, several limitations must be considered. iPSC-derived granulocytes and monocytes frequently exhibit not only incomplete maturation, but also altered cytokine secretion profiles and attenuated effector functions compared with their primary adult counterparts, and these differences have been shown to be highly protocol-dependent (19, 42, 72, 73). Moreover, line-to-line and batch-to-batch variability in differentiation efficiency necessitates the inclusion of multiple iPSC clones, isogenic controls, and standardized readouts.

Single-cell RNA sequencing (scRNA-seq) has become a useful tool for characterizing the identity and developmental states of iPSC-derived hematopoietic and myeloid cell populations. By mapping *in vitro* differentiation trajectories onto reference single-cell atlases of human hematopoiesis, this method can help position iPSC-derived cells along primitive and definitive hematopoietic programs, which is not always evident from cell surface immunophenotyping alone (138, 139). In the context of myeloid differentiation, temporal single-cell RNA/ATAC profiling has provided a reference map of *in vitro* human myelopoiesis (140), and scRNA-seq of iPSC-derived macrophages has been proposed as a quality-control tool to verify product identity and cellular composition before therapeutic application (141). In addition, computational approaches such as CellMap use reference single-cell datasets to decompose bulk RNA-seq from iPSC-derived cells and offer a practical approach to monitor cell-type composition and batch-to-batch variability in larger-scale applications (137).

A major historical limitation of iPSC-based hematopoietic differentiation has been the difficulty in generating sufficient numbers of engraftable definitive hematopoietic progenitors with sustained multilineage potential (142). Early attempts to address this used *in vivo* teratoma assays, in which human iPSCs are injected into immunodeficient mice and form complex tumors containing hematopoietic niches (143–145). In this setting, Philipp et al. demonstrated that human multilineage hematopoietic progenitors can arise in teratomas generated from iPSCs via EHT supported by co-injected human umbilical vein endothelial cells (HUVECs) in NSGS mice, which constitutively express human SCF, IL-3, and GM-CSF (146). However, teratoma-based hematopoiesis requires animal hosts, involves long and variable *in vivo* maturation, and depends on co-transplanted stromal/supporter cells, which makes it intrinsically low-throughput and poorly standardized for disease modeling, drug screening or generation of cells for clinical use (144, 146).

TF-based reprogramming strategies subsequently demonstrated that enforced expression of hematopoietic TFs (for example ERG, HOXA9, RORA) can endow pluripotent-derived or somatic cells with multilineage hematopoietic potential and in some settings support *in vivo* engraftment, but these approaches often rely on integrating vectors and show variable durability of HSC-like function (147, 148). Building on this concept, expandable hematopoietic progenitor cells (eHPCs) have been generated by transient or regulated expression of self-renewal-associated TFs, creating immortalized progenitor lines that retain disease-specific phenotypes while enabling high-throughput functional and drug screening, albeit with clear limitations for direct clinical translation (149, 150).

More recently, advances in differentiation conditions have improved the long-term engraftment capacity of iPSC-derived hematopoietic cells. A recent study by the Elefanty group demonstrated that guiding iPSCs through HOXA-patterned mesoderm to BMP4/VEGF-specified hemogenic endothelium, followed by VEGF withdrawal to promote EHT, generates CD34^+^ cells capable of robust, long-term multilineage engraftment in immunodeficient mice (151). These findings provide evidence that durable, repopulating hematopoietic cells can be derived from human iPSCs under fully defined conditions.

Complementing these advances, Frenz-Wiessner et al. recently reported the generation of complex bone marrow-like organoids from human iPSCs that self-organize into a three-dimensional niche comprising mesenchymal, endothelial, and multilineage hematopoietic compartments (152). This platform provides a structurally and molecularly bone marrow-like *in vitro* system to investigate human HSPC maintenance and differentiation in a physiologically relevant microenvironment.

Despite persistent challenges, iPSC-based hematopoietic systems have already transformed the study of human myelopoiesis. Patient-derived iPSCs representing pre-leukemic states, clonal hematopoiesis, and acute myeloid leukemia (AML) have been combined with directed differentiation into myeloid cells to reconstruct clonal evolution, uncover stage-specific vulnerabilities, and evaluate targeted therapies within defined human genetic backgrounds. Continued optimization of differentiation protocols toward better developmental fidelity, scalability, and definitive hematopoietic output is expected to expand the utility of iPSC-derived myeloid cells for mechanistic research, drug discovery, and, ultimately, regenerative applications.

## Modeling non-malignant myeloid cell disorders using iPSCs

iPSCs have become indispensable for modeling genetic disorders of myelopoiesis, including inherited bone marrow failure syndromes (IBMFS), myelodysplastic syndromes (MDS), and AML. Recent advances have further evolved these platforms from 2D differentiation protocols to 3D bone marrow organoids that better mimic the vascular and immune microenvironment (153). These human-centric models are accelerating the translation of mechanistic discoveries into clinical therapeutic strategies. The paramount advantage of iPSCs in hematology lies in their ability to preserve the specific human genomic context of the patient, enabling the study of disease mechanisms that are often obscured in animal models. Murine and zebrafish models frequently exhibit discordance with human phenotypes due to species-specific differences in genetic and mRNA/protein expression profiles, transcription factor binding, and immune ontogeny (154). A notable example is the failure of *Runx1*-mutant mice to fully replicate the clinical spectrum of human Familial Platelet Disorder with Propensity to Myeloid Malignancy (FPD/MM), a gap that *RUNX1*-mutant FPD/MM iPSC models have successfully bridged by recapitulating the human-specific defects in megakaryopoiesis (155).

**Figure 1:**
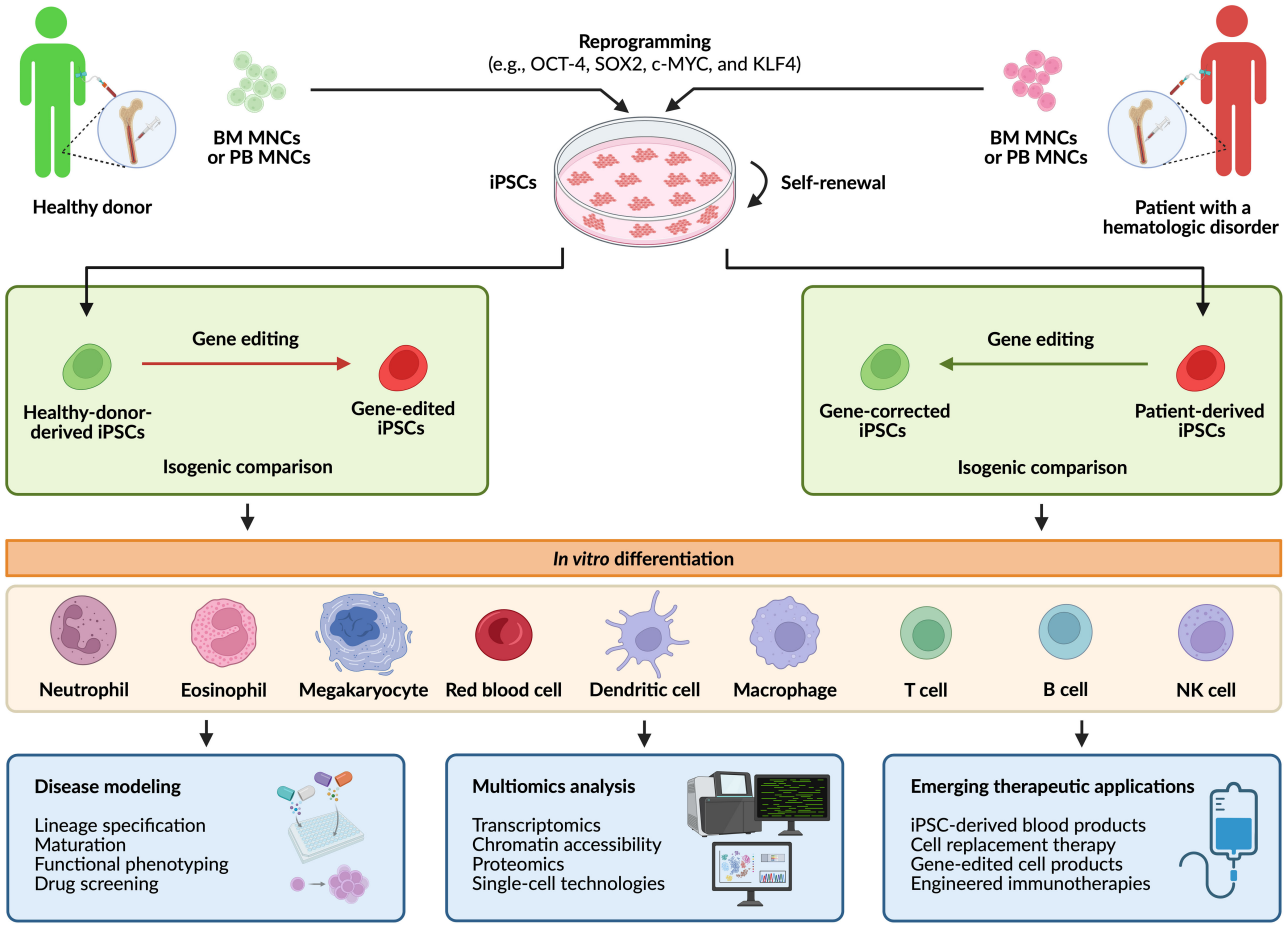
iPSC-based modeling of hematopoietic development in health and disease. Somatic cells (e.g., peripheral blood or bone marrow mononuclear cells) from healthy donors or patients with hematological disorders can be reprogrammed into induced pluripotent stem cells (iPSCs). Genome editing technologies (e.g., CRISPR/Cas9) enable generation of isogenic iPSC lines by introducing disease-associated variants into healthy-donor-derived iPSCs or correcting pathogenic mutations in patient-derived lines. Directed hematopoietic differentiation enables generation of diverse myeloid lineages (e.g., neutrophils, monocytes/macrophages, eosinophils, dendritic cells), as well as erythroid, megakaryocytic, and lymphoid cells, from the same iPSC source. These platforms support detailed mechanistic characterization of normal and pathological hematopoiesis, drug screening, and the development of gene- and cell-based therapies. BM, bone marrow; PB, peripheral blood; MNCs, mononuclear cells. Created with BioRender.

The utility of these models is maximized through CRISPR/Cas9-mediated genome editing to generate isogenic controls, effectively silencing genetic background noise and establishing direct genotype-phenotype causality. These models are generated either by introducing a pathogenic mutation into a wild-type background or by correcting a specific variant in patient-derived cells, thereby neutralizing the profound influence of genetic, epigenetic, metabolomic backgrounds on disease severity and drug response (156) (**Figure 1**).

The transformative impact of iPSC technology on IBMFS is best exemplified by recent advances in our understanding of CN. iPSC-derived hematopoietic progenitor and myeloid lineage cells from CN patients exhibited significantly diminished myeloid differentiation compared to healthy control iPSCs. This faithfully recapitulated the characteristic maturation arrest at the promyelocyte stage of granulopoiesis observed in CN patients (74). iPSC studies have also shed light on *HAX1*-related CN, uncovering distinct mechanisms involving reduced mitochondrial membrane potential (Δψ_m_) (157). Furthermore, Touw and colleagues have leveraged iPSC models to investigate the pre-leukemic evolution of CN, using patient-derived iPSCs with *ELANE* and *HAX1* mutations, particularly in the context of acquired *CSF3R* mutations (158). Their work demonstrated that truncated G-CSF receptors, which frequently arise in CN patients progressing to AML, fail to trigger proper differentiation signals and instead induce hyper-proliferation and a sustained pro-inflammatory state, thereby identifying key signaling nodes for therapeutic targeting (158). By providing a window into the earliest stages of hematopoietic defects, iPSC models have been instrumental in resolving the complex pathogenesis of *ELANE* mutations, the most common cause of CN (159, 160). The precise characterization of these disease-causing genotypes has subsequently enabled both the design of targeted gene-editing therapies for *ELANE*-related neutropenia and the refinement of patient-specific off-target assessments (160–163). Beyond CN, iPSC technology has been pivotal in elucidating the pathogenesis of Shwachman-Diamond syndrome (SDS). Patient-derived iPSC models have confirmed that *SBDS* mutations impair ribosome biogenesis – specifically the decreased ratios of the 80S and 60S subunits relative to the 40S subunit – thereby disrupting both hematopoietic and pancreatic differentiation (164). iPSC modeling of *GATA2* deficiency has also provided helpful insights. This is a rare genetic disorder characterized by the variable onset of a pleomorphic constellation of immune, hematologic, and lymphatic abnormalities. Linked to heterozygous mutations in *GATA2*, it leads to monocytopenia, B-cell and NK-cell lymphopenia, and dendritic cell deficiency as the disease progresses (165). Gene-edited isogenic iPSC models have demonstrated that *GATA2* haploinsufficiency leads to hematopoietic developmental defects (166).

iPSC technology has proved to be transformative in dissecting the pathophysiology of Diamond-Blackfan anemia (DBA). Models derived from patients with ribosomal protein mutations – most commonly in *RPS19* – have confirmed that the disease is driven by haploinsufficiency, which triggers a p53-mediated ribosomal stress response (167). Beyond confirming the p53-dependent cell cycle arrest, recent iPSC studies have resolved the enigma of tissue specificity in DBA. It has been demonstrated that the global reduction in ribosome levels selectively impairs the translation of specific transcripts with complex secondary structures, most notably the master erythroid transcription factor GATA1 (168). This translational inefficiency uncouples erythroid differentiation from proliferation, a defect that is fully reversible upon CRISPR/Cas9-mediated correction of specific point mutations in *RPS19* (e.g., c.191T>C and c.184C>T) (167). While these models are powerful tools for high-throughput ribosomal stress screens, their utility can be tempered by inter-line variability in erythroid output, which necessitates robust isogenic controls.

In contrast to the ribosomal stress of DBA, Fanconi anemia (FA) models highlight the challenges of inherent genomic instability. The generation of FA-iPSCs is notoriously difficult (155); the reprogramming process itself induces replication stress that FA-deficient cells cannot repair, often resulting in apoptosis or the selection of somatic variants. Consequently, successful reprogramming usually requires temporary complementation of the Fanconi pathway, transient knockdown of the p53-p21 axis, or culturing cells under hypoxic conditions (155, 169). Once established, these iPSCs serve as a unique platform for modeling hypersensitivity to DNA cross-linking agents (e.g., mitomycin C) and interstrand cross-links (ICLs). These models are currently driving the search for genoprotective agents, including scavengers of endogenous aldehydes (170, 171). However, the inherent fragility of these cells poses a continuous challenge, often leading to karyotypic evolution during long-term culture that can obscure drug response data.

Among monogenic red blood cell disorders, CRISPR/Cas9-edited iPSCs have become pivotal for modeling thalassemia and sickle cell disease. Unlike complex disorders, these diseases offer a clear target for gene correction (172). Patient-derived iPSCs edited to correct *HBB* mutations have demonstrated restored erythroid differentiation and maturation. Beyond the hematopoietic compartment, the systemic utility of stem cell therapies is being explored; combining iPSC-derived HSPCs with mesenchymal stem cells (MSCs) offers a potential avenue for alleviating multi-organ complications specifically associated with thalassemia, such as cirrhosis and cardiomyopathy, by leveraging the regenerative capacity of MSCs (172).

## Drug repurposing using iPSC models of non-malignant hematological conditions

**Figure 2:**
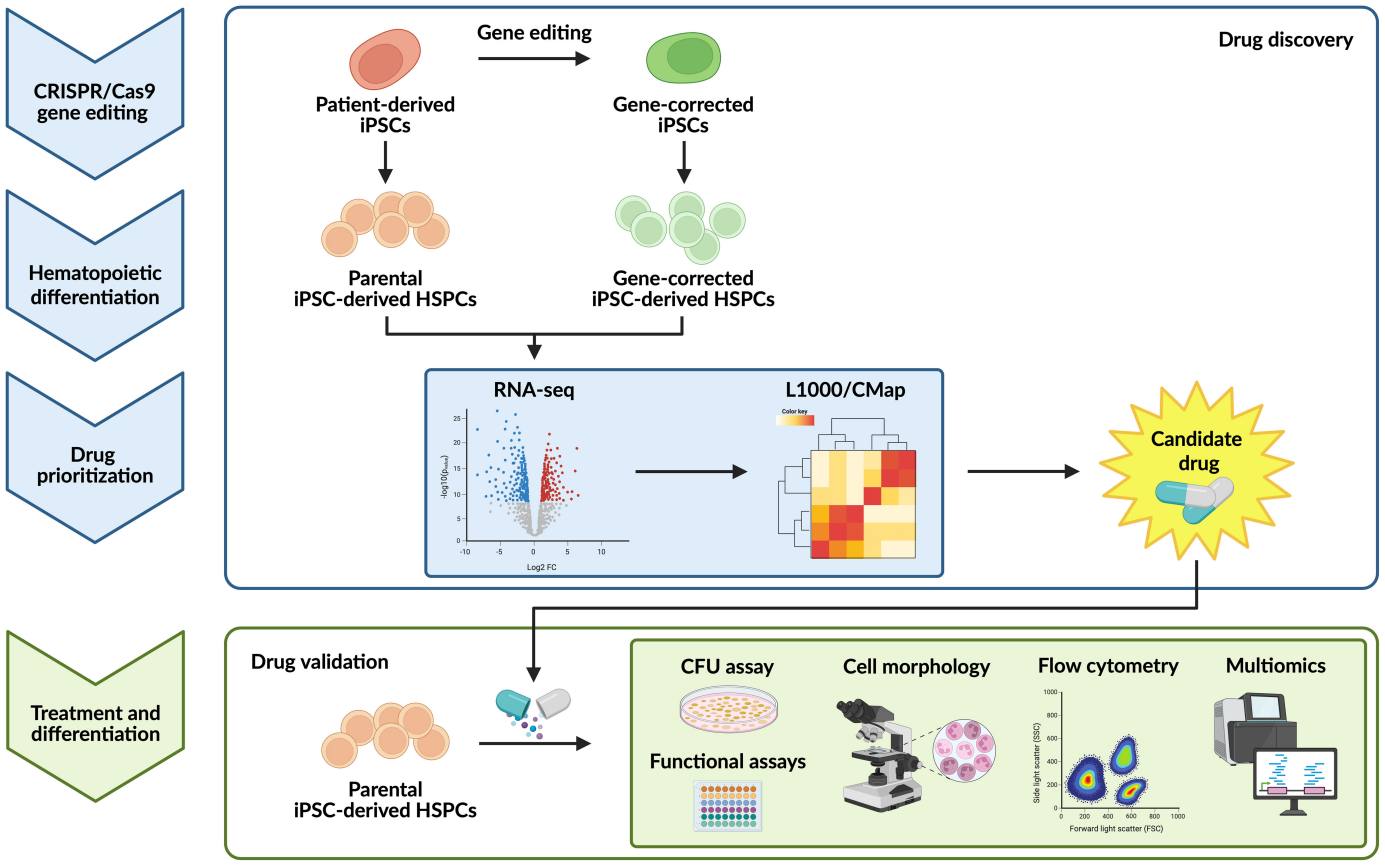
Integrated iPSC-based platform for drug discovery in myeloid disorders. Patient-derived iPSCs can be gene-corrected to generate isogenic lines. Following hematopoietic differentiation, patient-derived and gene-corrected iPSCs give rise to hematopoietic stem and progenitor cells (HSPCs), enabling downstream isogenic comparisons. Phenotypic assessment by flow cytometry, transcriptomic profiling, and complementary molecular analyses enable identification of disease-associated signatures, which can be interrogatedusing Connectivity Map (CMap) analysis to prioritize candidate therapeutic compounds. Drug validation can be subsequently performed in iPSC-derived HSPCs and their differentiated progeny. Differentiation readouts (e.g., morphology, colony-forming unit (CFU) assays, flow cytometry, multiomic profiling, lineage-specific functional assays) enable systematic evaluation of phenotypic rescue and therapeutic efficacy in a preclinical setting. Created with BioRender.

Beyond elucidating pathogenesis of hematological diseases, iPSC platforms have emerged as powerful engines for drug repurposing, accelerating the identification of therapeutic candidates by enabling high-throughput phenotypic screens directly in human disease-relevant cells (**Figure 2**). This approach bypasses the species-specific metabolic discrepancies that often plague murine drug trials. A cornerstone of modern computational repurposing is the integration of iPSC-derived transcriptomic data with large-scale perturbation databases, most notably the Connectivity Map (CMap) (173). Expanding upon earlier iterations, the next generation CMap leverages the high-throughput L1000 platform. This cost-effective assay measures mRNA abundance of a reduced set of ~978 “landmark” genes, which were carefully selected to capture the majority of cellular transcriptional variance. By utilizing established gene-expression correlations, the platform can then computationally infer the expression levels of the remaining ~81% of the transcriptome with high accuracy (173, 174). This massive library now encompasses over one million gene expression profiles representing the cellular responses of human cell lines to thousands of small molecules and genetic perturbations. The standard workflow operates on the principle of signature mimicking or reversion. First, RNA-seq is performed on the patient-specific iPSC model and its isogenic control to define a quantitative “disease signature” – a ranked list of differentially expressed genes driven by the specific mutation. This signature is then queried against the L1000 database using algorithms that search for inverse connectivity. The goal is to identify compounds that induce a transcriptional profile negatively correlated with the disease state – effectively canceling out the pathogenic gene expression program (174). This approach has proven particularly powerful when combined with interactive visualization tools (e.g., L1000FWD), which allow clustering of drugs by their mechanism of action, thereby identifying novel therapeutic classes that mimic the transcriptional effects of known rescue agents or genetic knockdowns.

In the context of bone marrow failure, *in silico* transcriptomic analysis of *ELANE*-mutant iPSCs identified flavopiridol, an FDA-approved cyclin-dependent kinase (CDK) inhibitor, as a potent drug for rescuing granulopoiesis with potential therapeutic applications. By targeting the aberrant transcriptional machinery linked to cell cycle arrest and mimicking the transcriptional activity of C/EBPα, flavopiridol restored granulopoiesis – a finding subsequently validated in zebrafish models, where it demonstrated efficacy without significant toxicity (175).

Drug repurposing is also being leveraged to refine the iPSC differentiation protocols themselves. Recently, small-molecule screens identified dobutamine, a standard inotropic agent, as a modulator of EHT. Mechanistically, dobutamine inhibits Yes-associated protein (YAP) activity, a critical negative regulator of EHT, thereby significantly enhancing the yield of iPSC-derived HSPCs (176).

## Modeling pre-leukemia and myeloid neoplasms using iPSCs

In leukemia research, scientists historically relied on immortalized leukemic cell lines and genetically engineered mouse models. The modeling of human AML and MDS using primary material has been difficult due to the refractoriness of myeloid blasts to *ex vivo* expansion and the inability of animal models to fully capture the complex architecture of human hematologic neoplasms. The derivation of iPSCs from cells of patients with hematologic malignancies has offered a transformative solution. By reprogramming leukemic blasts to pluripotency, researchers can capture the patient’s germline and somatic mutations in a state that allows indefinite expansion. This approach is particularly valuable when leukemia arises in patients with rare IBMFS. It addresses the common challenge of obtaining sufficient cell numbers for the study of pre-leukemic conditions (such as clonal hematopoiesis) and MDS. These iPSCs can then be differentiated back into hematopoietic lineages, recreating the disease “in a dish”.

The promising results were initially achieved in reprogramming of myeloproliferative disorders (MPD). The first reports on the generation and characterization of human iPSCs derived from MPD were published in 2009 and 2010, describing successful reprogramming of chronic myeloid leukemia cells harboring the *BCR::ABL1* translocation (177) and peripheral blood CD34^+^ cells from patients with polycythemia vera (PV) and primary myelofibrosis (PMF) harboring the somatic *JAK2* p.V671F mutation (178).

However, the application of iPSC technology to MDS and AML has proven significantly more challenging. The first report describing reprogramming of MDS-iPSCs from two patients with chromosome 7q deletion was published by Kotini et al. in 2015 (179). In 2017, the same research group successfully reprogrammed primary human AML cells into iPSCs and pioneered the “stepwise” modeling approach, utilizing gene editing to reconstruct the evolutionary history of myeloid malignancies in human cells (7). By generating a spectrum of iPSCs ranging from pre-leukemia and low-grade MDS to high-grade MDS and secondary AML, they established an iPSC-based platform capable of tracking disease progression from a healthy state to overt malignancy. Another landmark study in the iPSC field demonstrated that reprogramming primary AML cells resets their methylation landscape; however, subsequent hematopoietic differentiation triggers the reacquisition of leukemic pathobiology (6). This indicates that the underlying leukemic mutations drive the return of aberrant DNA methylation signatures. Intriguingly, both studies reported that AML-iPSC-derived HSPCs were capable of robust engraftment in immunodeficient mice (6, 7).

Several studies have highlighted significant technical hurdles in applying iPSC technology to myeloid malignancies; specifically, certain leukemia-associated DNA lesions represent a major barrier to the successful reprogramming of leukemic cells (180–183) (**Table 1**). For instance, Yamasaki et al. were able to generate only cytogenetically normal iPSCs from the bone marrow (BM) of an AML patient carrying the high-risk der(7)t(7;13) translocation (181). Similarly, attempts to reprogram AML cells harboring the t(8;21) translocation (182) or a complex karyotype (183) have been unsuccessful. With further development of iPSC reprogramming and maintenance protocols, successful reprogramming has been achieved for leukemic cells carrying the most common AML driver mutations, such as *KRAS* mutations (6, 7, 184), *FLT3* internal tandem duplication (6, 65, 185), *MLL* translocations (6, 65), *PML*-*RARA* fusion (65), and mutations in splicing factor genes *SF3B1*, *SRSF2*, *U2AF1* (65, 150, 186–189) and others. As a result, Kotini et al. recently demonstrated that most leukemic subtypes can, in fact, be reprogrammed to pluripotency (65). iPSC-based modeling of stage-specific leukemogenesis using CRISPR/Cas9-mediated introduction of (pre-)leukemia-associated mutations in CN iPSCs has identified *BAALC* as a critical oncogene driving the transition from CN to overt leukemia. This progression, characterized by a block in myeloid differentiation and enhanced progenitor survival, suggests that targeting the *BAALC* signaling axis could provide a precision therapeutic window for preventing leukemic transformation in high-risk CN patients (74).

**Table 1:** AML- and MDS-associated genetic alterations in iPSC models of hematologic diseases.

Gene/Locus	Genetic alteration	Reprogramming efficiency	Key findings from iPSC-based studies	Primary studies
*KMT2A (MLL)*	t(9;11) and t(11;19) rearrangements	High/Easy	Reacquisition of leukemic properties upon differentiation; myeloid-restricted phenotype; sensitivity to DOT1L inhibitors; robust xenograft engraftment.	Chao et al. (2017) (6); Kotini et al. (2023) (65)
*KRAS/NRAS*	p.G12D, p.G13D	High (often captured as subclones)	Enhanced proliferation; *KRAS* p.G13D confers sensitivity to MEK inhibitors. RAS-mutant leukemia stem cells drive venetoclax resistance through monocytic differentiation.	Chao et al. (2017) (6); Sango et al. (2024) (184); Wang et al. (2021) (186)
*NPM1*	Frameshift	Very low/Failed (strong negative selection)	Reprogramming typically yields normal clones or preleukemic clones carrying only a *DNMT3A* mutation.	Kotini et al. (2023) (65);
*SF3B1*	p.K700E, p.G742D, p.H662Q	Moderate (wild-type cells often outcompete mutant cells)	Impaired erythropoiesis; ring sideroblast formation; mis-splicing of *TMEM14C* and *ABCB7*.	Clough et al. (2022) (245); Asimomitis et al. (2022) (188)
*EZH2*	Loss-of-function	Successful	Exacerbation of erythroid defects in *SF3B1*-mutant MDS.	Clough et al. (2022) (245)
*SRSF2*	p.P95L	Successful	Dysplastic morphology; altered splicing of *EZH2*; sensitivity to splicing inhibitors (e.g., E7107).	Chang et al. (2018) (150); Wang et al. (2021) (186); Kotini et al. (2023) (65)
*U2AF1*	p.S34F, p.Q157R	Successful	AML-derived iPSCs successfully generated transplantable cells with leukemic characteristics.	Kotini et al. (2023) (65)
*FLT3*	Internal tandem duplication (ITD)	High/Easy	Less differentiated leukemic population; functional redundancy with RAS mutations in transformation.	Kotini et al. (2023) (65); Chao et al. (2017) (6)
*ASXL1*	C-terminal truncations	Successful	Impaired myeloid differentiation; an MDS/CHIP-like state; insufficient to induce a frank leukemic phenotype unless combined with *SRSF2* and *NRAS* mutations.	Wang et al. (2021) (186); Kotini et al. (2017) (7)
*TET2*	Loss-of-function	Successful	Impaired myeloid differentiation; DNA hypermethylation; often acts as an initiating event in models of clonal evolution.	Beke et al. (2020) (246); Kotini et al. (2023) (65)
*PML::RARA*	t(15;17)	High/Easy	Successfully modeled in iPSC lines representing acute promyelocytic leukemia; robust reprogramming efficiency.	Kotini et al. (2023) (65)
*RUNX1*	Somatic and germline mutations	Low/difficult for somatic mutations; successful for germline mutations	FPD (haploinsufficiency): megakaryocytic maturation defect and platelet dysfunction. FPD (dominant-negative): promotion of myelopoiesis, genomic instability. CN/AML: blocked granulocytic differentiation, hyperproliferation, and *BAALC* upregulation. With *SF3B1* mutations (MDS): expansion of HSC/MPPs; myeloid lineage bias; inflammatory signaling. AML iPSC model: *RUNX1* is required for leukemic stem cell self-renewal and engraftment..	Sakurai et al. (2014) (247); Iizuka et al. (2015) (248); Antony-Débré et al. (2015) (249); Dannenmann et al. (2021) (74), Wesely et al. (2020) (250), Sarchi et al. (2025) (187);
*CSF3R*	Nonsense	Successful	Context-dependent proliferation and clonal advantage; impaired granulocytic differentiation; reduced CFU-G and CFU-GM colony formation; induction of proinflammatory signaling.	Dannenmann et al. (2021) (74); Olofsen et al. (2023) (158)
del(7q)	7q deletion	Successful (moderate)	Profound block in differentiation; reduced clonogenic potential and viability; spontaneous genetic correction. In SDS models, it further impairs defective hematopoiesis and reduces the production of CD34^+^ and CD45^+^ cells; cells show selective sensitivity to niflumic acid, a COX-2 inhibitor.	Kotini et al. (2015) (179); Chang et al. (2018) (150); Ruiz-Gutiérrez et al. (2019) (251)
*DNMT3A*	p.R882H/C	Successful	*DNMT3A* p.R882H/C mutations are necessary for AML initiation but are largely dispensable for disease maintenance.	Kotini et al. (2023) (65); Köhnke et al. (2026) (252)
*IDH2*	p.R140Q, p.R172K	Successful	Low to no engraftment in NSGS mice.	Wang et al. (2021) (186); Kotini et al. (2023) (65)
*STAG2*	Loss-of-function	Successful (modeled in an *SF3B1*-mutant context)	In *SF3B1*-mutant cells, STAG2 loss blocks differentiation and downregulates myeloid gene programs (divergent from RUNX1 effects).	Sarchi et al. (2025) (187)
del(5q)	5q deletion	Difficult; successful when co-occurring with *TP53* mutations	Identified *LMNB1* as a critical haploinsufficient driver gene located on 5q. In the context of *TP53* mutations, del(5q) contributes to an impaired DNA-damage response and increased genomic instability.	Hsu et al. (2019) (183); Reilly et al. (2022) (253)
*TP53*	Missense	Variable	Increased genomic instability of *TP53*-mutant iPSCs with del(5q).	Hsu et al. (2019) (183); Kotini et al. (2023) (65)
*WT1*	Frameshift	Subclonal loss	*WT1* mutations were found to be subclonal. Consequently, the resulting iPSC clones were derived from the *WT1* wild-type subclones.	Kotini et al. (2023) (65)

The ability of iPSCs to produce sufficient cell numbers for small- to medium-scale compound screening has led to broad application of iPSC-derived HSPCs for pharmacological evaluation and target discovery in myeloid malignancies. Wang et al. demonstrated that repurposing cyclosporin A to target the interaction between peptidylprolyl isomerase like 2 (PPIL2) and p53 axis offers a novel strategy for *JAK2*-mutant myeloproliferative neoplasms (190). Furthermore, recent iPSC screens have uncovered that MDS clones harboring mutations in the cohesin complex genes (e.g., *STAG2, SMC3*) are exquisitely sensitive to PARP inhibitors. This vulnerability, driven by a defect in replication fork stability, suggests that PARP inhibitors – traditionally used in BRCA-mutant solid tumors – could be repurposed for specific subsets of MDS/AML (191). Additionally, a screen of ~2,000 compounds in iPSCs derived from MDS patients with 7q deletion of 7q (del(7q)) identified niflumic acid, a COX-2 inhibitor, as a compound that can selectively inhibit the mutant clones (150). Furthermore, iPSC models allowed for the identification of therapeutic vulnerabilities in *SF3B1*-mutated MDS. Using HSPCs derived from *SF3B1*-mutant iPSCs, Sarchi et al. demonstrated the efficacy and selectivity of the CHK1 inhibitor prexasertib *in vivo* (192).

Using the *in silico* drug repurposing platform CMap, CN/AML-iPSC-derived leukemia cells were found to be susceptible to treatment with CMPD1, a small-molecule inhibitor of phospho-MK2 that effectively and selectively eliminates leukemic blasts while sparing healthy progenitors (74). The identification of prexasertib (192) and MK2 inhibitors (74) demonstrated the practical applicability of iPSCs in a “bedside-to-bench-to-bedside” functional search of potential therapies against myeloid malignancies. Similarly, in iPSCs representing early pre-leukemic stages (e.g., harboring *ASXL1* or *SRSF2* mutations), IRAK1/4 and UBE2N inhibitors were identified as effective agents against early-stage disease clones, suggesting a potential strategy for prevention of disease progression (186). Similarly, Ruiz-Gutiérrez et al. used iPSCs derived from patients with SDS to model leukemogenic progression by engineering del(7q). The authors demonstrated that while TGF-β pathway inhibition rescued hematopoiesis in the initial SDS stage, it was ineffective in cells harboring del(7q) (251).

In addition, iPSC models have been instrumental in assessing chemotherapy resistance. Thus, iPSC lines capturing both *KRAS*-mutated and *KRAS* wild-type subclones from the same patient demonstrated differential sensitivities. *KRAS*-mutant subclones were sensitive to MEK inhibitors (e.g., trametinib, PD98059), whereas *KRAS* wild-type subclones were resistant to MEK inhibition but sensitive to DOT1L inhibitors (6). Recent applications of iPSC models have also elucidated mechanisms of resistance to venetoclax, a BCL2 inhibitor (184).

Several key limitations and challenges of using iPSCs to study myeloid malignancies remain to be solved. These include the resistance of AML and MDS cells to reprogramming, particularly cells with specific mutations (e.g., in *NPM1*) or complex chromosomal abnormalities. The generated cells typically display a “fetal-like” immaturity, failing to fully represent adult hematopoiesis. Additionally, researchers working with iPSCs face hurdles such as clonal selection bias and the poor *ex vivo* proliferation of MDS and AML cells, which severely hampers iPSC generation efficiency.

The current consensus is that while iPSC-derived leukemic cells reliably preserve the major genetically defined leukemic clones and their relative hierarchy, prolonged culture and downstream manipulations inevitably introduce clonal drift (193, 194). The reprogramming and selection of rare or late-arising subclones is particularly challenging, as these populations are easily overlooked without extensive colony screening. Additionally, clones with specific fitness or epigenetic features may be preferentially reprogrammed or fail to reprogram at all (195). Recent studies have sought to address these limitations. For instance, patient-derived AML iPSCs generated with “complete capture of mutational burden” (CCoMB) retained the mutational and cytogenetic spectrum of the primary leukemias across all major genetic AML subgroups (65). In practice, iPSCs are faithful models of specific leukemic clones rather than exact, time-stable replicas of the full *in vivo* clonal ecosystem.

Other challenges include the lack of physiological selective pressure *in vitro* and differentiation blocks induced by certain driver mutations at early developmental stages. Finally, the derivation and differentiation processes themselves are labor-intensive, time-consuming, and prone to significant line-to-line variability.

Despite these biological and technical hurdles, the unique advantages of iPSC technology continue to drive substantial progress in the field of experimental hemato-oncology. The modeling of myeloid neoplasms using iPSCs has overcome early skepticism to become a powerful alternative and complement to traditional cell lines, primary patient cells, and *in vivo* mouse and zebrafish models. By accurately recapitulating the stepwise acquisition of leukemogenic mutations and revealing novel therapeutic vulnerabilities, iPSC-derived models of myeloid malignancies have become indispensable. As gene editing and differentiation protocols continue to refine, iPSC-based platforms promise to deliver the next generation of targeted therapies for myeloid malignancies.

## Therapeutic applications of iPSC-derived cells in hemato-oncology

So far, the clinical application of iPSC-derived blood cells has largely been in preclinical development, with only a few advanced trials to date. iPSC-derived blood cells have the potential to be used in blood cell replacement therapy and in oncological settings. Advantages of iPSC-derived blood cell products over donor-derived cells include an almost unlimited supply of such cells from a single iPSC line, improved safety (e.g., no risk of viral transmission from donors), and the ability to genetically manipulate them – for example, using CRISPR/Cas9-mediated gene editing – to enhance the functions of iPSC-derived blood cells, such as by extending their circulation time or enabling therapeutic loading or correction of genetic defects. The relatively straightforward genetic manipulation of iPSCs enables the production of cells with a knockout of surface protein complexes – human leukocyte antigen (HLA) classes I or II (*B2M, TAP2, CIITA, CD74*), which are typically recognized by T lymphocytes, as well as the NK-cell-activating ligands MICA and MICB (196, 197). This is combined with overexpression of PD-L1/L2 to further suppress recognition by T cells (198) or of CD47 to avoid phagocytosis by macrophages (199), as well as with reintroduction of HLA-E and/or HLA-G as a key T-cell and NK-cell evasion strategy (197, 200–202). These allogeneic, or “off-the-shelf”, iPSCs have broad immune compatibility, allowing scalable cell therapies without the need for patient-specific sources of iPSCs.

Nakamura et al. established the *ex vivo* generation of platelets from immortalized iPSC-derived megakaryocyte cell lines (imMKCL) for transfusion in patients with thrombocytopenia (203). This has resulted in the first-in-human clinical trial using autologous iPSC-derived platelets (iPSC-PLTs) in a patient with transfusion-refractory aplastic anemia (204–206). In this patient, iPSC-PLTs were detected in circulation. However, no increase in platelet count was observed following transfusion with iPSC-PLTs, suggesting the need for further system optimization. Using the same imMKCL system, the same group also succeeded in generating HLA class I-depleted iPSC-PLTs. These can serve as a single off-the-shelf product that evades recognition by the recipients’ immune system (207). iPSC-derived platelets can also be used as targeted carriers of factor VIII (FVIII) for patinets with hemophilia, or of chemotherapeutic drugs for homing to and targeting tumors.

Varga et al. have recently reported the successful large-scale production of transfusion-ready iPSC-derived red blood cells (iRBCs), using a feeder-free, GMP-compatible system (208). The generated iRBCs were functional, but resembled a fetal phenotype, which may be acceptable for therapeutic use, since partial expression of fetal hemoglobin (HbF) is considered a therapeutic approach for patients with sickle cell disease and β-thalassemia.

iPSC-derived neutrophils (iNeus) retained key functions such as phagocytosis, migration, and the formation of neutrophil extracellular traps (NETs). They displayed strong antimicrobial activity *in vitro* against *Klebsiella pneumoniae, Pseudomonas aeruginosa, Escherichia coli*, and *Staphylococcus aureus* and enhanced the survival of mice with neutrophil dysfunction in lethal bacterial infections (209).

iPSC-derived macrophages (iMacs) also have high potential for clinical translation. iMacs can be used to treat pulmonary diseases such as pulmonary proteinosis. When administered intratracheally to *Csf2rb*-deficient mice, human iMacs can differentiate into tissue-resident alveolar macrophages and alleviate disease (210, 211). *In vitro* and *in vivo* studies have also shown the effectiveness of iMacs for bacterial clearance and as immunotherapies against bacterial airway infections (123, 212). Ackermann et al. have demonstrated that the intrapulmonary transplantation of bioreactor-derived human iMacs rescued mice from acute infections of the lower respiratory tract caused by *Pseudomonas aeruginosa* (123). Another study revealed that the adoptive transfer of human iMacs in immunodeficient mice had potent antimicrobial effects against clinical isolates of methicillin-sensitive and methicillin-resistant *Staphylococcus aureus* (212). Similarly, the differentiation of iPSC-derived monocytes into microglia cells has been demonstrated to possess significant therapeutic potential for neurodegenerative diseases (213, 214). Intravenous delivery of iPSC-derived mononuclear phagocytes improved performance in hippocampus-dependent cognitive tasks, reduced neuroinflammation in aging mice, and enhanced cognition in both young and aging 5×FAD mouse models of Alzheimer’s disease (213). The brains of 2- and 6-month-old 5×FAD mice showed significant reductions in amyloid-β (Aβ) plaques, astrogliosis, and microglial activation after treatment with extracellular vesicles (EVs) from human iPSC-derived microglia (214). iPSC-derived microglia can also deliver therapeutic proteins, depending on the underlying pathology. For instance, they can express the Aβ-degrading enzyme neprilysin under the control of a plaque-responsive promoter in an experimental mouse model of Alzheimer’s disease (215). iMacs also represent a promising platform for anti-cancer cell therapy. They can be engineered to enhance tumor killing by expressing chimeric antigen receptors (CAR-iMacs), in combination with blockade of checkpoint receptors such as the sialic acid-binding immunoglobulin-like lectins Siglec-5 and Siglec-10, as well as metabolic reprogramming via aconitate decarboxylase 1 (ACOD1) depletion (216–221). To our knowledge, there are currently no active clinical trials for CAR-iMacs.

The generation of HSCs from iPSCs for transplantation in patients with acquired or inherited bone marrow failure or leukemia remains far from clinical translation due to the inherent differences between iPSC-derived HSCs and their primary bone marrow counterparts. Unlike native HSCs, iPSC-derived HSCs do not naturally undergo the transition from primitive to definitive hematopoiesis, which helps explain the difficulty of achieving robust engraftment. Only a few studies have reported engraftable iPSC-derived hematopoietic cells, with recent pioneering work by the Elefanty group demonstrating the adaptation of culture protocols that support their efficient generation (151).

Taken together, iPSCs represent a valuable source for clinical products, but they still have limitations, including the high costs, long production times of *ex vivo* generated of iPSC-derived blood products, and significant line-to-line variability. As a result of the inherent differences between iPSC-derived blood cells and their natural counterparts, not all functions can be reproduced in iPSC-derived products. Additionally, primary blood cells and their iPSC-derived counterparts exhibit distinct epigenetic profiles, compounded by the emergence of HSC-like populations from reprogramming and incomplete epigenetic resetting during differentiation. Furthermore, iPSCs frequently acquire chromosomal aberrations through the selection of fast-proliferating clones – a risk that demands rigorous monitoring of iPSC lines approved for clinical production. Extended culture periods introduce the risk of “culture-acquired” aneuploidy or dominant-negative *TP53* mutations. Therefore, rigorous quality control has become an essential prerequisite for publication and clinical translation (222). Finally, for applications moving toward cell therapy, tumorigenicity remains a paramount concern. The potential for residual undifferentiated pluripotent cells to form teratomas poses a significant safety risk. Current mitigation strategies include the rigorous purification of CD34^+^ progenitors by magnetic-bead sorting and the engineering of suicide-gene safeguards (e.g., inducible caspase-9). These systems allow for the remote, chemical elimination of the graft should a malignant clone emerge, thereby providing a necessary safety brake for clinical applications (223).

## Engineering the microenvironment for advanced human hematopoietic and myeloid models using organ-on-chip technology: current platforms and opportunities

Both for modelling purposes and for regenerative medicine applications, the sourcing and generation of fully functional and human hematopoietic and myeloid cells is crucial. Yet, during the last two decades, it has become increasingly clear that, beyond obtaining physiologically relevant human cells, accurately replicating the native, dynamic microenvironments the cells experience *in vivo* is essential for *in vitro* applications. Microphysiological systems, particularly Organ-on-Chip (OoC) technology, have emerged as powerful tools for replicating *in vivo* characteristics of the cellular microenvironment and organ-level functionality (224, 225). Over the past several years, numerous Bone-Marrow-on-Chip systems were introduced to recreate bone marrow niches, investigate cell biology, model pathological conditions, and conduct drug screening assays (226); yet, none of them so far combines human iPSC and OoC technologies. Using murine primary cells, Torisawa et al. introduced one of the pioneering models by first generating an artificial bone containing living marrow in mice through the subcutaneous implantation of an osteoinductive material housed within a cylindrical polydimethylsiloxane (PDMS) carrier (227). After eight weeks of *in vivo* maturation, the engineered bone-marrow construct was explanted and transferred into a PDMS-based microfluidic device for subsequent *in vitro* culture. Following four days under microfluidic conditions, self-renewing, multipotent hematopoietic stem cells were successfully maintained. The platform was further applied as an *in vitro* model to investigate radiation-induced toxicity. One of the first models of the human bone marrow was developed by Sieber et al.: a hydroxyapatite-coated zirconium oxide scaffold seeded with human primary mesenchymal stromal cells and cord blood-derived multipotent HSPCs was incorporated into a dynamically perfused transwell platform (228). This engineered system enabled long-term *in vitro* maintenance of HSPCs for up to 28 days, preserving their primitive CD34^+^CD38^−^ phenotype as well as their multilineage differentiation potential, including granulocyte, erythrocyte, macrophage, and megakaryocyte colony formation. Importantly, the platform supported the establishment of a three-dimensional microenvironment that recapitulated key structural and functional features of the native bone marrow niche (228). More recently, a refined version of the model, integrating commercially available CD34^+^ HSPCs from adult human bone marrow, was utilized for safety profiling of T-cell bispecific as well as transferrin receptor-binding antibodies (229).

With particular emphasis on the vascular component, Glaser et al. employed a microfluidic platform comprising two hexagonal chambers to generate a three-dimensional *in vitro* model of human Bone-Marrow-on-Chip that incorporates both perivascular and endosteal compartments within a dynamically perfused vascular network (230). The system is based on human cord blood-derived CD34^+^ HSPCs as well as endothelial colony-forming cells and a human fetal osteoblast cell line. It reproduces essential features of bone marrow physiology, supporting the preservation and lineage-specific differentiation of HSPCs, enabling the release of CD66b^+^ neutrophils, and exhibiting compartment-dependent responses to doxorubicin and G-CSF (230). 

Looking forward, the convergence of advances in iPSC-derived human hematopoietic and myeloid systems outlined in the previous sections with the precise microenvironmental control afforded by OoC technologies is set to fundamentally transform disease modeling and enable the systematic development and rigorous evaluation of next-generation therapeutic strategies.

## Clinical translation barriers of iPSC-derived hematopoietic and myeloid cells

Despite considerable progress in generating immature hematopoietic cells and various myeloid cell types from iPSCs, several barriers limit clinical translation.

A major concern is the immunogenicity of allogeneic iPSC-derived cells, which can elicit immune responses upon transplantation. Complete suppression of the receptors and pathways that sense and trigger immune responses is unlikely to be feasible: immune cells derived from such immune-edited HSCs may lose some immunoregulatory functions (199), and leukemogenic transformation of the engrafted iPSC-derived HSCs might escape immune recognition and clearance by immune cells.

Genomic instability and the associated risk of tumorigenicity represent further important safety concerns. They may be driven by: (i) pre-existing mutations in the parental somatic cells, which become clonally expanded during reprogramming (231), (ii) reprogramming-induced mutations, largely attributable to replicative stress and reactive oxygen species generated by the reprogramming factors themselves (232, 233), and (iii) acquired mutations or recurrent karyotypic aberrations that accumulate during prolonged *in vitro* iPSC culture (61, 234, 235).

Manufacturing under GMP conditions also presents substantial challenges. For clinical application, iPSCs and their hematopoietic cell derivatives should be produced under GMP-compliant conditions, which substantially increases manufacturing costs. Additionally, iPSC lines require regular assessment of genomic and chromosomal integrity, and clone- or passage-dependent variability may result in batch-to-batch deviations that further complicate standardization and clinical use (110, 231, 236).

Incomplete reprogramming may cause persistent epigenetic abnormalities in early-passage iPSCs, including residual donor-cell methylation signatures that bias differentiation toward the parental lineages (36, 37), possible abnormal X-chromosome inactivation in female lines, and occasional loss of imprinting (237, 238). More recent work has demonstrated that donor memory attenuates with passaging and that clone-to-clone epigenetic variability, driven partially by donor genetic background, is at least as important as origin-specific memory in shaping differentiation potential (110, 239).

Finally, the absence of robust protocols for the GMP-compliant, large-scale generation of engraftable HSCs remains a major obstacle to clinical application. Substantial progress has been made in achieving long-term multilineage engraftment of iPSC-derived HSCs in mice, either by transduction with seven transcription factors – *ERG, HOXA5, HOXA9, HOXA10, LCOR, RUNX1*, and *SPI1* (240), or by stepwise HOXA-patterned iPSC differentiation (151). However, no protocol has yet been described that reliably enables clinical-scale, xeno-free, GMP-compliant manufacture of engraftable multilineage HSCs from iPSC lines for transplantation. This limitation reflects the persistent difficulty of directing iPSC-derived hematopoiesis toward adult-like definitive HSCs rather than fetal- or yolk-sac-like hematopoietic programs (111, 151). Addressing this bottleneck, alongside the immunological, tumorigenic, epigenetic and manufacturing challenges, will be essential for translating iPSC-derived hematopoietic and myeloid cell products into routine clinical practice (241, 242).

## Conclusion and future perspectives: toward a “Clinical trial in a dish”

As we move beyond the foundational era of iPSC technology, the field is undergoing a paradigm shift from simple 2D reductionism to complex, systems-level modeling. The future of hematological disease modeling lies in the convergence of stem cell biology with bioengineering and artificial intelligence (AI). While 2D cultures have been instrumental, they lack the spatial architecture and mechanical forces of the native bone marrow. Future advancements will increasingly rely on 3D bone marrow organoids (152, 243) and microfluidic OoC systems (227). These platforms engineer a physiological niche by co-culturing HSPCs with mesenchymal stromal cells, osteoblasts, and vascular networks within a 3D matrix. Crucially, these systems recapitulate the vascular shear stress and oxygen gradients absent in static dishes – factors known to regulate HSC quiescence and drug resistance (153). By mimicking the protective “sanctuary” sites of the marrow, these models will be pivotal for studying niche-mediated drug resistance in leukemia and testing therapies designed to mobilize malignant stem cells from their dormant state (227). The sheer volume of data generated by these models necessitates a computational revolution. Combining high-content screening with single-cell multiomics (scRNA-seq, scATAC-seq, and proteomics) enables unprecedented resolution of cellular heterogeneity. AI and machine learning algorithms are now being trained on these high-dimensional datasets to predict drug responses and identify non-obvious therapeutic targets. By creating “digital twins” of patient-derived organoids, AI can simulate thousands of drug combinations in silico before validating the top candidates *in vitro*, drastically compressing the timeline of drug discovery (244). Ultimately, these technologies converge on the goal of “N-of-1” precision medicine. For rare IBMFS, where patient cohorts are too small for traditional randomized clinical trials, iPSCs offer a unique solution. They allow for the generation of “virtual cohorts” – unlimited numbers of patient-specific cells that can undergo massive parallel testing. This facilitates rapid drug repurposing, reducing the reliance on costly *de novo* drug development and accelerating the delivery of life-saving therapies to patients with orphan diseases.

In summary, iPSC technology has graduated from a novelty to a cornerstone of modern hematology and hemato-oncology. By bridging the gap between genomic discovery and clinical application, iPSC-based platforms are poised to deliver on the promise of personalized medicine, transforming the way we understand, diagnose, and treat hematological disorders.
